# Functional Divergence in Shrimp Anti-Lipopolysaccharide Factors (ALFs): From Recognition of Cell Wall Components to Antimicrobial Activity

**DOI:** 10.1371/journal.pone.0067937

**Published:** 2013-07-04

**Authors:** Rafael Diego Rosa, Agnès Vergnes, Julien de Lorgeril, Priscila Goncalves, Luciane Maria Perazzolo, Laure Sauné, Bernard Romestand, Julie Fievet, Yannick Gueguen, Evelyne Bachère, Delphine Destoumieux-Garzón

**Affiliations:** 1 Ecologie des Systèmes Marins Côtiers, UMR5119, Centre National de la Recherche Scientifique, Institut Français de Recherche pour l’Exploitation de la Mer, Institut de la Recherche pour le Développement, Université Montpellier 1, Université Montpellier 2, Montpellier, France; 2 Laboratory of Immunology Applied to Aquaculture, Department of Cell Biology, Embryology and Genetics, Federal University of Santa Catarina, Florianópolis SC, Brazil; French National Centre for Scientific Research - Université de Toulouse, France

## Abstract

Antilipopolysaccharide factors (ALFs) have been described as highly cationic polypeptides with a broad spectrum of potent antimicrobial activities. In addition, ALFs have been shown to recognize LPS, a major component of the Gram-negative bacteria cell wall, through conserved amino acid residues exposed in the four-stranded β-sheet of their three dimensional structure. In penaeid shrimp, ALFs form a diverse family of antimicrobial peptides composed by three main variants, classified as ALF Groups A to C. Here, we identified a novel group of ALFs in shrimp (Group D ALFs), which corresponds to anionic polypeptides in which many residues of the LPS binding site are lacking. Both Group B (cationic) and Group D (anionic) shrimp ALFs were produced in a heterologous expression system. Group D ALFs were found to have impaired LPS-binding activities and only limited antimicrobial activity compared to Group B ALFs. Interestingly, all four ALF groups were shown to be simultaneously expressed in an individual shrimp and to follow different patterns of gene expression in response to a microbial infection. Group B was by far the more expressed of the *ALF* genes. From our results, nucleotide sequence variations in shrimp ALFs result in functional divergence, with significant differences in LPS-binding and antimicrobial activities. To our knowledge, this is the first functional characterization of the sequence diversity found in the ALF family.

## Introduction

Anti-lipopolysaccharide factors (ALFs) are antimicrobial peptides (AMPs) only found in marine chelicerates (horseshoe crabs) and crustaceans, which exhibit a potent antimicrobial activity against a broad range of microorganisms. The spectrum of the antimicrobial activity of ALFs covers a large number of Gram-positive and Gram-negative bacteria, filamentous fungi as well as enveloped viruses [1]–[5]. Initially isolated from the hemolymph of the horseshoe crabs *Tachypleus tridentatus* (ALF-T) and *Limulus polyphemus* (ALF-L) [6], ALFs were later identified in penaeid shrimps by transcriptomic-based approaches [7]–[9]. ALFs are known as highly cationic polypeptides of about 100 residues with a hydrophobic N-terminal region. The horseshoe crab ALF-L and the shrimp ALF*Pm*3, from *Penaeus monodon*, share a similar three-dimensional structure, consisting in three α-helices packed against a four-stranded β-sheet [10], [11]. Two conserved cysteine residues are involved in an intramolecular disulfide bridge which delimits the central β-hairpin.

ALF homologues have been described in many crustacean species, especially in penaeid shrimp (for review see [12], [13]). In *P. monodon*, for which studies in ALF characterization are relatively advanced, six different ALF variants (ALF*Pm*1 to 6) have been identified [14], [15]. Based on amino acid sequence comparisons, these variants were classified into three main groups: Group A (ALF*Pm*1-2), Group B (ALF*Pm*3-5) and Group C (ALF*Pm*6), which are encoded by distinct genomic loci [14], [16]. Beyond differences in gene organization (two or three introns), shrimp ALF Groups A to C are differentially transcribed among shrimp tissues. Indeed, ALF*Pm*3 and ALF*Fc* from *Fenneropenaeus chinensis* (Group B) are expressed in hemocytes, while *Mj*ALF2 from *Marsupenaeus japonicus* and ALF*Pm*6 from *P. monodon* (Group C) are expressed in different shrimp tissues [14], [17]–[19].

**Figure 1:**
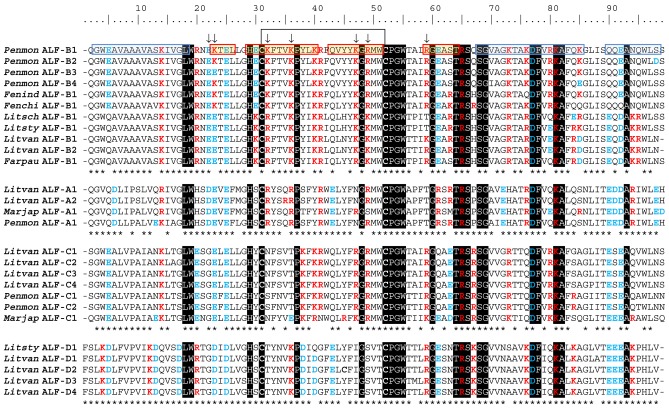
Amino acid sequence alignments of mature polypeptides of the four ALF groups (Group A, B, C and D) found in penaeids. Asterisks (*) indicate residues conserved in each specific group of ALF sequences. Residues conserved in all ALF sequences are highlighted in black. The conserved cysteine bridge is indicated. Residues involved in LPS-binding of Group B ALFs [11] are indicated by arrows. α-helices and β-strands identified in the three-dimensional structure of *Penmon* ALF-B1 (PDB entry 2JOB) [11] are indicated by blue and red boxes, respectively.

As observed for other cationic AMPs from amphibians and fishes [20], [21], both horseshoe crab and shrimp ALFs from Groups B and C bind and neutralize LPS [6], [10], [19], [22], a major component of the outer membrane of Gram-negative bacteria. In horseshoe crab ALFs, positively-charged residues recognizing the lipid A moiety of LPS would be located in the β-hairpin stabilized by the disulfide bridge [10]. Such a β-hairpin structure is preserved in cyclic synthetic peptides designed on the central-most residues of the β-hairpin, hydrophobic residues at position 44 and 46 of the horseshoe crab ALF sequence playing a crucial role in stabilizing the hydrophobic face of the β-hairpin [23]. In such cyclic synthetic peptides, the positively-charged residues bind in an exothermic reaction with the negative charges of LPS and Lipid A, which further shows the crucial role of the β-hairpin in its interaction with the LPS acyl chains [24]. Like the native horseshoe crab ALF, the recombinant shrimp ALF*Pm*3 binds to Lipid A and the most probable LPS-binding site involves six positively-charged residues and one negatively-charged amino acid located in the cysteine-stabilized β-hairpin and in the two neighboring β-strands (**Figure 1**) [11]. Recombinant ALF*Pm*3 was also proved to bind to lipoteichoic acid (LTA), a major cell wall component of Gram-positive bacteria [19]. However, the amino acid residues involved in LTA-binding are still unknown. Interestingly, the ability of shrimp ALFs to bind to bacterial cell wall components, such as LPS and LTA, has been suggested to be associated with their antibacterial activities [19].

Studies on ALFs have mainly focused on the highly active and cationic ALFs belonging to Group B (for review see [12], [13]), which have been shown to be essential in the protection of shrimp against different microbial infections [7], [14], [25], [26]. However, while many sequences of shrimp ALFs have been described, little attention has been paid to the functional consequences of the ALF sequence diversity in terms of biochemical properties and biological activities.

Herein, taking advantage of the identification of a novel group of shrimp ALFs with unique anionic properties and displaying an incomplete LPS binding site (Group D ALFs), we have undertaken a study of shrimp ALF functional diversity. Our phylogenetic and functional data show that shrimp ALFs have evolved as four functionally diverse groups, among which some have shaped important motifs for interacting with the cell wall components of bacteria and inhibiting bacterial growth. Those groups show different patterns of expression in response to infection. We provide here the first evidence of functional divergence in shrimp ALFs. In order to standardize the current classification of this diverse family of AMPs, we propose here a common nomenclature for shrimp ALFs.

## Materials and Methods

### Molecular Cloning and Sequence Analysis

For the molecular cloning of a cationic ALF member (Group B) in the blue shrimp *Litopenaeus stylirostris*, specific primers were designed based on consensus nucleotide sequences of ALFs from different shrimp species (**Table 1**). Total RNA was extracted from hemocytes by homogenization with Trizol reagent (Invitrogen) following the manufacturer’s instructions. Following heat denaturation (70°C for 5 min), reverse transcription was performed using 1 µg of total RNA with 50 ng/µl oligo(dT)_12–18_ in a 20-µl reaction volume containing 1 mM dNTPs, 1 unit/µl of RNAseOUT Ribonuclease and 200 units/µl M-MLV reverse transcriptase in reverse transcriptase buffer according to the manufacturer’s instructions (Invitrogen). PCR reactions were conducted in a 25-µl reaction volume using 1 µl of synthesized complementary DNA (cDNA) as template. PCR conditions were as follows: 30 cycles of 94°C for 1 min, 55°C for 1 min, 72°C for 1 min and a final elongation step of 72°C for 10 min. The amplification products were cloned into a pCR 2.1 TA cloning vector using a TA cloning kit (Invitrogen). The positive recombinant clones were identified by colony PCR and were sequenced in both directions.

**Table 1:** Nucleotide sequence of primers used in this study.

Primer name	Forward primer (5′−3′)	Reverse primer (5′−3′)	
Primers for cDNA amplification			
*LstyB1*	AGTAACTTTCCTAGTTTAGA		CTGGCGCGGGAAAGGCCTA
Primers for protein expression			
*LstyD1rp*	GCGCGAATTCATGTTTTCGCTAAAAGACCTTTTTG		ATATATGTCGACTTATACAAGGTGTGGTTTGGC
Primers for quantitative Real-Time PCR (RT-qPCR)			
*Lvanrpl40qt*	GAGAATGTGAAGGCCAAGATC		TCAGAGAGAGTGCGACCATC
*LvanAqt*	CTGATTGCTCTTGTGCCACG		TGACCCATGAACTCCACCTC
*LvanBqt*	GTGTCTCCGTGTTGACAAGC		ACAGCCCAACGATCTTGCTG
*LvanCqt*	ATGCGAGTGTCTGTCCTCAG		TGAGTTTGTTCGCGATGGCC
*LvanDqt*	TGTGTTGGTTGTGGCACTGG		CAACGAGGTCAATGTCACCG

Annotated ALF sequences from penaeid shrimp were methodically collected from publicly accessible databases (GenBank, EMBL) and used for the search of homologous sequences among EST sequences from the NCBI database. Homology searches were performed using BLAST at NCBI. The multiple alignments were generated using the MAFFT alignment program (http://align.bmr.kyushu-u.ac.jp/mafft/online/server/). Prediction of signal peptide was performed with the SignalP program (http://www.cbs.dtu.dk/services/SignalP/). The phylogenetic analysis based on the amino acid sequences of shrimp and chelicerate ALFs and scygonadins (outgroup) was performed using the Neighbour-Joining method with the software MEGA version 4.0 [27]. Bootstrap sampling was reiterated 1,000 times. The uncovered sequence phylogenies were used to define distinct groups of ALF sequences.

### Synthetic and Recombinant Peptides

Peptide amino acid sequences are shown in **Table 2**. Synthetic peptides *Penmon* ALF-B1 β-hairpin, *Litsty* ALF-B1 β-hairpin and *Litsty* ALF-D1 β-hairpin were obtained by Fmoc chemistry and purchased from Genepep S.A. (Montpellier, France). Recombinant *Penmon* ALF-B1 (also referred to as rALF*Pm*3) was over-expressed and purified as previously described [4]. Recombinant *Litsty* ALF-D1 was expressed in *Escherichia coli* Rosetta (DE3) as an N-terminal His6-tagged fusion protein using the pET-28a system (Novagen). The open reading frame of interest was PCR-amplified from cDNA samples derived from hemocytes of *L. stylirostris* using specific primers (**Table 1**), a Met-coding trideoxynucleotide was incorporated 5′ of each cDNA and cloned in-frame with the N-terminal His6 in the EcoRI/SalI sites of pET-28a. Expression of recombinant *Litsty* ALF-D1 was performed as described previously for oyster defensins [28], [29]. The purification procedure started with affinity chromatography by incubating bacterial cell lysates with TALON® metal affinity resin (Clontech) at a ratio of 25∶1 (v/v) in 6 M guanidine HCl, 50 mM sodium phosphate, 300 mM NaCl, 5 mM imidazole, (pH 8.5) for 4 h at 4°C with gently agitation. Then, resin was washed twice by decantation in 6 M guanidine HCl, 50 mM sodium phosphate, 300 mM NaCl (pH 8.5), and fusion proteins were eluted by decantation with two column volumes of 6 M guanidine HCl, 50 mM sodium phosphate and 1 M imidazole (pH 6.4). The eluate was desalted on a reversed phase Sep-pak C-18 cartridge (2O cc). Separation was performed with a step gradient of 10% and 80% of acetonitrile (ACN) containing 0.05% Trifluoroacetic acid (TFA). The 80% ACN fraction containing the peptide mixture was then frozen and lyophilized. The methionine residue introduced at the peptide N-terminus was subjected to CNBr cleavage as described previously [28]. The cleaved fusion peptide mixture was then directly folded at pH 8.1 in a refolding solution containing 0.1 M NaHCO_3_, 3 mM reduced glutathione and 0.3 mM oxidized glutathione in the presence of 2 M urea and 25% N,N-dimethylformamide, at room temperature for 72 h. The peptide mixture containing *Litsty* ALF-D1 was then purified on a preparative reversed-phase Zorbax 300 SB-C8 column with a biphasic gradient of 0–32% ACN over 10 min and 32–47% ACN over 50 min at a flow rate of 3 ml/min. Peptide purity was assessed by MALDI-TOF-MS and SDS-PAGE analysis.

**Table 2:** Peptide sequences.

Peptide	Amino acid sequence	Mass (kDa)	p*I*
***Penmon*** ** ALF-B1** **(GenBank: ABP73289)**	QGWEAVAAAVASKIVGLWRNEKTELLGHE**C**KFTVKPYLKRFQVYYKGRMW**C**PGWTAIRGEASTRSQSGVAGKTAKDFVRKAFQKGLISQQEANQWLSS	11.05	9.95
***Litsty*** ** ALF-D1** **(GenBank: AAY33769)**	FSLKDLFVPVIKDQVSDLWRTGDIDLVGHS**C**TYNVKPDIQGFELYFIGSVT**C**PGWTTLRGESNTRSKSGVVNSAVKDFIQKALKAGLVTEEEAKPHLV	10.81	6.10
***Penmon*** ** ALF-B1** **(Synthetic β-hairpin)**	G**C**KFTVKPYLKRFQVYYKGRMW**C**G	2.96	9.93
***Litsty*** ** ALF-D1** **(Synthetic β-hairpin)**	G**C**TYNVKPDIQGFELYFIGSVT**C**G	2.61	4.37
***Litsty*** ** ALF-B1** **(Synthetic β-hairpin)**	G**C**RFTVKPYIKRIQLHYKGKMW**C**G	2.91	10.04

Sequences of *Penmon* ALF-B1 and *Litsty* ALF-D1 are shown without signal peptide, in their expected mature form. Conserved cysteine residues are in bold face. Molecular weight (kDa) and predicted isoelectric point (p*I*) of the recombinant peptides and synthetic ALF β-hairpins are indicated on the right.

### Antimicrobial Assays

The antimicrobial activity of peptides was assayed against different microorganisms: the Gram-positive bacteria *Aerococcus viridans* (CIP 104 074), *Micrococcus luteus* (CIP 5345 and IBMC collection), *Bacillus megaterium* (CIP 6620), *Brevibacterium stationis* (CIP 101 282) and *Staphylococcus aureus* (ATCC 65 38), the Gram-negative bacteria *E. coli* SBS363, *Salmonella enterica* (CIP 5858), *Vibrio alginolyticus* (CIP 103336 T), *V. harveyi* E22, *V. anguillarum* V62, *V. penaeicidae* AM101 and *V. nigripulchritudo* SFn1 and the fungi *Candida albicans*, *Fusarium oxysporum*, *Rhizopus stolonifer*, *Septoria nodorum* and *Botrytis cinerea*. Minimum inhibitory concentrations (MICs) were determined in triplicate by the liquid growth inhibition assay based on the procedure described in [30]. MIC values are expressed as the lowest concentration tested that causes 100% of growth inhibition (µM). Poor Broth (PB, 1% bactotryptone, 0.5% NaCl w/v, pH 7.5) was used as a culture medium for bacteria. It was supplemented with 0.5 M NaCl for marine bacteria (*B. stationis* and *Vibrio* strains). When needed, sea salts (20 µM KCl, 5 µM MgSO_4_, 1.5 µM CaCl_2_ final concentration) were also added to the culture medium. Brain heart infusion (BHI) broth (Becton Dickinson) was used for *Aerococcus viridans*. Potato Dextrose Broth (Difco) at half strength was used for cultures of filamentous fungi, while Sabouraud Dextrose Broth (Difco) was used for yeast (*C. albicans*) cultures. Growth was monitored spectrophotometrically at 620 nm on a Multiscan microplate reader (Labsystems).

### LPS-binding Assay

The LPS-binding properties of recombinant ALFs were tested using the QCL-1000 *Limulus* amoebocyte lysate (LAL, Lonza). Briefly, *E. coli* LPS at 1 EU/ml was incubated in a 1∶1 (v/v) ratio with 0.001 to 5 µM recombinant ALFs or polymixine B (Sigma), used as a control. After 15 min at 37°C, 1 vol of *Limulus* amoebocyte lysate was added to the reaction. After 10 min at 37°C, the chromogenic LAL substrate was added. The reaction was stopped after a 6 min-incubation by adding 1 vol of 25% acetic acid. Absorbance was read at 405 nm.

### Animals, Tissue Collection and Immune Challenge

*Litopenaeus vannamei* adult shrimp (10±2 g) were obtained from the Laboratory of Marine Shrimp (Federal University of Santa Catarina, Brazil). Shrimp were acclimated at 23°C for at least 72 h before experimental infection. Then, shrimp received in intramuscular injection (50 µl) of *Fusarium solani* (5×10^6^ spores/animal) or sterile sea water (SSW), as injury control. The standardization of the experimental infections and the preparation of the fungal inoculum were performed as previously described [31]. Three groups of three shrimp were used in each experimental condition. Hemolymph from unchallenged and challenged shrimp was collected at 24 h and 48 h from the ventral sinus into a precooled modified Alsever solution (MAS: 27 mM sodium citrate, 336 mM NaCl, 115 mM glucose, 9 mM EDTA, pH 7.0). Hemocytes were isolated by centrifugation to discard plasma (700×*g* for 10 min at 4°C) and directly processed for RNA extraction.

### Real-time Quantitative PCR (RT-qPCR) Analysis of Gene Expression

Total RNA was extracted from hemocytes using Trizol reagent according to manufacturer instructions (Invitrogen). RNA was then treated with DNase I (Invitrogen) for 15 min at room temperature and inactivated by heat, 10 min at 65°C. After sodium acetate precipitation, quantity and quality of total RNA were determined using a NanoDrop spectrophotometer (*NanoDrop* Technologies) and agarose gel electrophoresis, respectively. Following heat denaturation of 1 µg of total RNA (65°C for 5 min), first strand synthesis was carried out using ImProm-II™ reverse transcriptase following the manufacturer protocol (Promega).

Gene expression of ALF members was analyzed by RT-qPCR on a LightCycler 480 System (Roche) in a final volume of 6 µl containing 5 mM MgCl_2_, 0.5 µM of each primer, 3 µl of reaction mix (LightCycler 480 SYBR Green I Master 2X) and 1 µl of each reverse transcribed RNA (diluted at 1∶19 in DNase/RNase-free distillated water). Primer sequences are listed in **Table 1**. RT-qPCR assays were submitted to an initial denaturation step of 10 min at 95°C followed by 40 cycles of denaturation at 95°C for 10 s, annealing at 57°C for 20 s and extension time at 72°C for 25 s. RT-qPCR assays were performed in triplicate, and standard curves were generated using six two-fold serial dilutions from a pool of all cDNAs (in DNase/RNase-free distillated water) to determine primer pair efficiencies (E) according to the equation: E* = *10^[–1/slope]^. Only primer pairs with efficiencies of 2±0.2 were selected for RT-qPCR relative quantification using the 2^−ΔΔCq^ method [32] with the *L. vannamei* ribosomal protein L40 (*Litvan-rpl40*, GenBank: FE077602) as reference gene. Data were analyzed using the LightCycler 480 software version 1.5.0.39 and the 2^nd^ derivative max algorithm. Statistical significance was determined using Student’s *t*-test between conditions and differences were considered when *p*<0.05.

## Results

### Identification of Novel Members of the ALF Antimicrobial Peptide Family in Shrimp

All annotated ALF sequences available for penaeid shrimp in publicly accessible databases were collected (**Table S1**) and used to screen non-annotated nucleotide sequences from EST projects using BLAST at NCBI. New EST sequences homologous to ALFs were identified, which included 28 sequences from the Pacific white leg shrimp *L. vannamei* (GenBank: FE109538; FE087264; FE088625; FE105941; FE153599; FE052210; FE176556; FE176555; FE058235; FE155445; FE079082; FE088301; FE090668; FE078559; FE079755; FE151634; FE152534; FE110967; FE115964; FE098450; FE156649; FE155982; FE152063; FE116643; FE115660; FE183080; FE092417; FE109539) and 4 sequences from the black tiger shrimp *P. monodon* (GenBank: DW678002; HO000126; GO080476; GW993385). Only full-length coding sequences (CDS) were kept for subsequent analyses. These data were completed by PCR amplification and sequencing of a novel ALF sequence obtained from the blue shrimp *L. stylirostris* (GenBank: KC346373).

From those 63 sequences, shrimp ALFs appear to be encoded as precursors starting with a 22–26 residue hydrophobic signal peptide followed by a 69–98 residue mature polypeptide containing two conserved cysteine residues (**Table S1**; **Figure 1**). Mature ALFs differed in terms of size with calculated molecular weights ranging from 7.69 to 11.52 kDa (**Table S1**; **Figure 1**), and more remarkably, in terms of electrostatic properties. Indeed, in addition to the known highly cationic ALFs (calculated p*I* ≥ 9.5), we found here a series of anionic ALFs (calculated p*I* ≤ 6) which differed from the previously described Group A ALFs [16], [33]. Interestingly, both cationic and anionic ALFs were shown to be present in a same shrimp species (**Table S1**).

### Shrimp ALFs Cluster into Four Distinct Groups with Contrasted Overall Charges

Phylogenetic analyses were performed with ALF sequences from penaeid shrimp and from the horseshoe crabs *T. tridentatus* (TACTR_ALF: P07087; TACTR2_ALF: AAK00651), *L. polyphemus* (LIMPO_ALF: P07086) and *Carcinoscorpius rotundicauda* (CARRO_ALF: CK086627). A total of 42 sequences were analyzed. A phylogenetic tree was generated from the sequence alignments with scygonadins [34], another two cysteine-containing AMP family found in crustaceans, used here as an outgroup.

**Figure 2:**
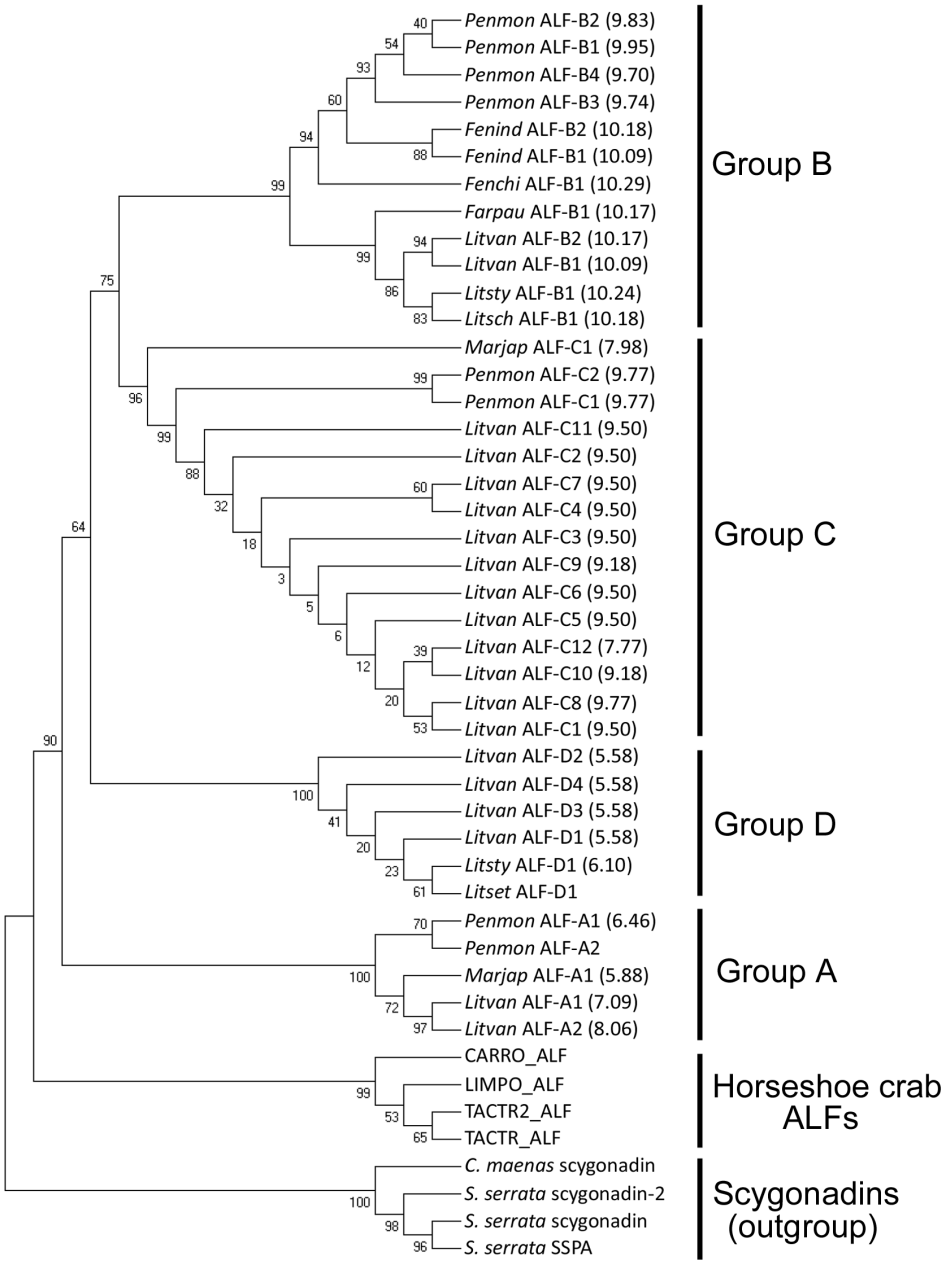
Penaeid shrimp ALFs cluster into four groups. ALF sequences from penaeid shrimps and horseshoe crabs, and scygonadins from crabs (outgroup) were aligned with MAFFT alignment program prior to phylogenetic analysis. The tree was constructed using the Neighbour-Joining method (Pairwise deletion) in MEGA 4. Bootstrap sampling was reiterated 1,000 times. Sequences included in analyses were the following: (i) Shrimp ALFs: black tiger shrimp *Penaeus monodon* (*Penmon* ALF-A1 or ALF*Pm*2: ABP73291; *Penmon* ALF-A1 or ALF*Pm*1: ABP73290; *Penmon* ALF-B1 or ALF*Pm*3: ABP73289; *Penmon* ALF-B2 and -B3 [47]; *Penmon* ALF-B4: ADC32520; *Penmon* ALF-C1 or ALF*Pm*6: ADM21460; *Penmon* ALF-C2 or ALF*Pm*6: AER45468), kuruma prawn (*Marjap* ALF-A1 or *Mj*ALF2: BAH22585; *Marjap* ALF-C1 or M-ALF: BAE92940), fleshy prawn *Fenneropenaeus chinensis* (*Fenchi* ALF-B1 or ALF*Fc*: AAX63831), Indian prawn *F. indicus* (*Fenind* ALF-B1: ADE27980; *Fenind* ALF-B2: ADK94454), pink shrimp *Farfantepenaeus paulensis* (*Farpau* ALF-B1 or ALF*Fpau*: ABQ96193), Atlantic white shrimp *Litopenaeus setiferus* (*Litset* ALF-D1: BE846661), Pacific white leg shrimp *L. vannamei* (*Litvan* ALF-A1 or *Lv*ALF1: EW713395; *Litvan* ALF-A2: FE087264; *Litvan* ALF-B1 or ALF*Lv*3: ABB22833; *Litvan* ALF-B2 or ALF*Lv*2: ABB22832; *Litvan* ALF-C1: FE153599; *Litvan* ALF-C2: FE176556; *Litvan* ALF-C3: FE058235; *Litvan* ALF-C4: FE079082; *Litvan* ALF-C5: FE088301; *Litvan* ALF-C6: FE078559; *Litvan* ALF-C7: FE079755; *Litvan* ALF-C8: FE105941; *Litvan* ALF-C9: FE090668; *Litvan* ALF-C10: FE052210; *Litvan* ALF-C11: FE088625; *Litvan* ALF-C12 or: *Lv*ALF2: EW713396; *Litvan* ALF-D1: FE152534; *Litvan* ALF-D2: FE151634; *Litvan* ALF-D3: FE110967; *Litvan* ALF-D4: FE115964), Southern white shrimp *L. schmitti* (*Litsch* ALF-B1 or ALF*Lsch*: ABJ90465) and blue shrimp *L. stylirostris* (*Litsty* ALF-B1: AGH32549; *Litsty* ALF-D1: AAY33769); (ii) Horseshoe crab ALFs: Chinese horseshoe crab *Tachypleus tridentatus* (TACTR_ALF: P07087; TACTR2_ALF: AAK00651), Atlantic horseshoe crab *Limulus polyphemus* (LIMPO_ALF: P07086) and Southeast Asian horseshoe crab *Carcinoscorpius rotundicauda* (CARRO_ALF: CK086627); (iii) Scygonadins (outgroup): giant mud crab *Scylla serrata* (*S serrata* scygonadin: AAW57403; *S serrata* scygonadin-2: ABI96918; *S serrata* SSAP: ABM05493) and green crab *Carcinus maenas* (*C. maenas* scygonadin: DY307310).

Sequences of shrimp ALFs clustered into four groups distinct from horseshoe crab ALFs (**Figure 2**). A first group named Group A [16], which was the more distant from the other three groups, corresponded to sequences similar to ALF*Pm*2 from *P. monodon* (referred to here as *Penmon* ALF-A1). Groups B [16] and C [14] corresponded to sequences similar to ALF*Pm*3 (*Penmon* ALF-B1) and ALF*Pm*6 (*Penmon* ALF-C1), respectively. Finally, a new group of shrimp ALFs, termed here Group D, clustered separately and contained sequences similar to those found *L. setiferus* [8] and *L. stylirostris* [7] (**Figure 2**). Interestingly, Group A to C gather sequences from occidental and Asian shrimp species, whereas Group D gathers sequences from occidental shrimp only. Scygonadins (outgroup) showed no clear relationship with shrimp ALFs and clustered in a distinct group from both shrimp and horseshoe crab ALFs.

Remarkably, the four ALF groups showed contrasted calculated isoelectric points (p*I*). Peptides from Group C and B were cationic (calculated p*I* = 7.77–9.77) and highly cationic (calculated p*I = *9.70–10.29), respectively. Peptides from Group A ranged from anionic to cationic (calculated p*I* = 5.88–8.06). Finally, peptides from Group D were very anionic (calculated p*I* = 5.58–6.10).

Based on both our sequence and phylogenetic analyses (**Figures 1** **and** **2**; **Table S1**), we propose here a uniform nomenclature for shrimp ALFs, similar to that earlier proposed for penaeidins, another family of antimicrobial peptides from penaeid shrimp [35]. Like the penaeidin (PEN) nomenclature, it includes: (i) the name of the penaeid shrimp in six letters and in italics (three for the genus and three for the species) followed by a space, (ii) the abbreviation “ALF” (for anti-lipopolysaccharide factor) followed by a hyphen and (iii) a number for the identification inside the group (*e.g.* ALF*Pm*3 is now referred to as *Penmon* ALF-B1). The determination of shrimp ALF Groups is based on the amino acid sequences of the mature peptide (**Figure 1**; **Table S1**).

### Group D ALFs Display an Incomplete LPS-binding Site and have Impaired LPS-binding Activities

Alignment of the amino acid sequences of the most anionic shrimp ALFs (Group D, p*I* ≤ 6.10) with the most cationic ones (Group B, p*I* ≥ 9.95). showed that 18% of the amino acids were identical. In particular, the two cysteines delimiting the central β-hairpin are conserved in all sequences. Other residues were group specific (**Figure 1**). Interestingly, we observed that the Group D ALFs were lacking most of the residues proposed to be involved in LPS-binding of Group B ALFs [11] (**Figure 1**).

**Figure 3:**
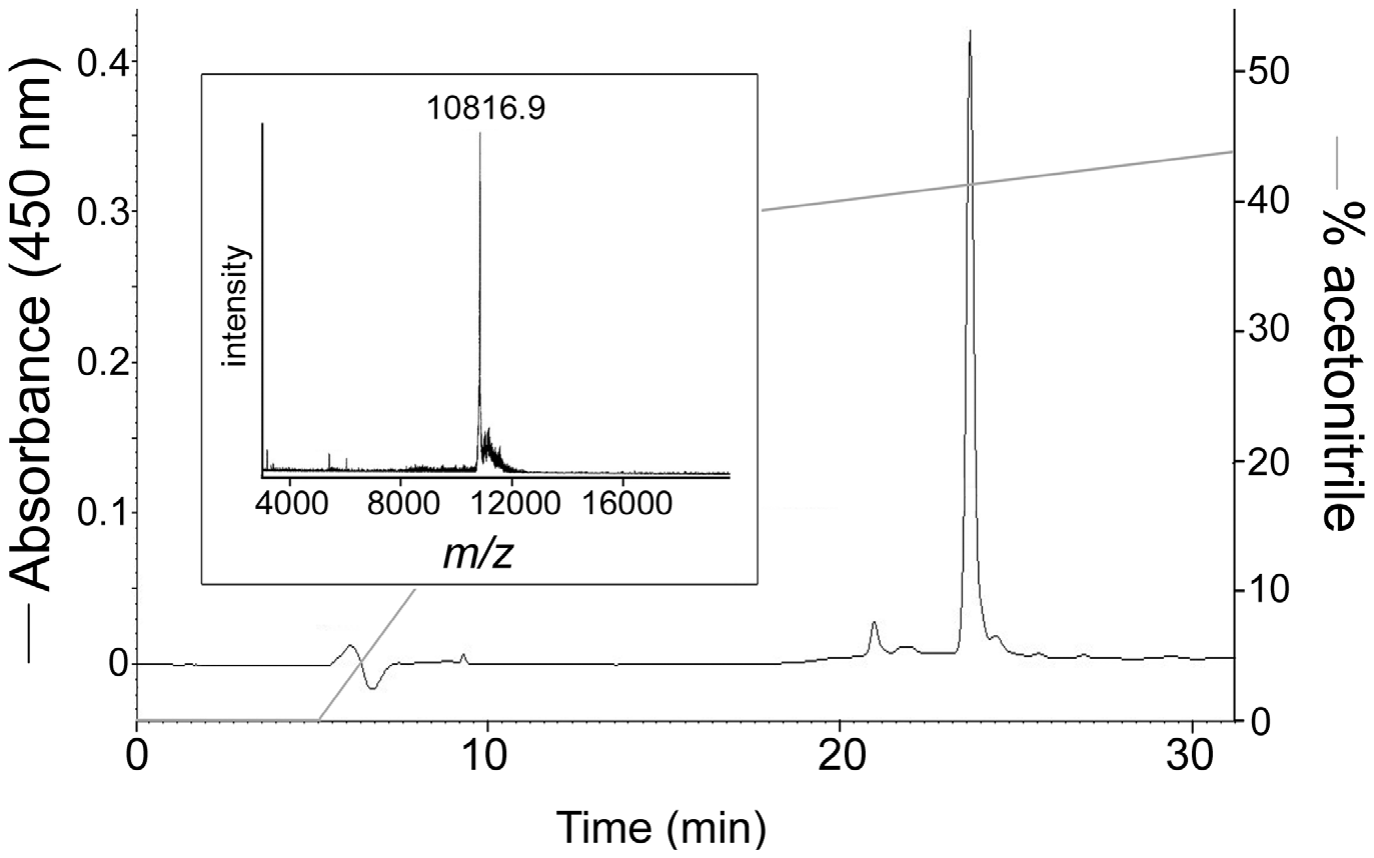
Purification of recombinant *Litsty* ALF-D1. HPLC profile of recombinant *Litsty* ALF-D1 on a C18 UP5NEC 25QS column. The acetonitrile percentage corresponding to the biphasic gradient is shown as a *grey line*. Recombinant *Litsty* ALF-D1 was observed as an absorbance peak (black line) eluted at 42% acetonitrile. The MALDI-TOF-MS spectrum of the collected peak (*inset*) showed a single mass at *m/z = *10816.9.

**Figure 4:**
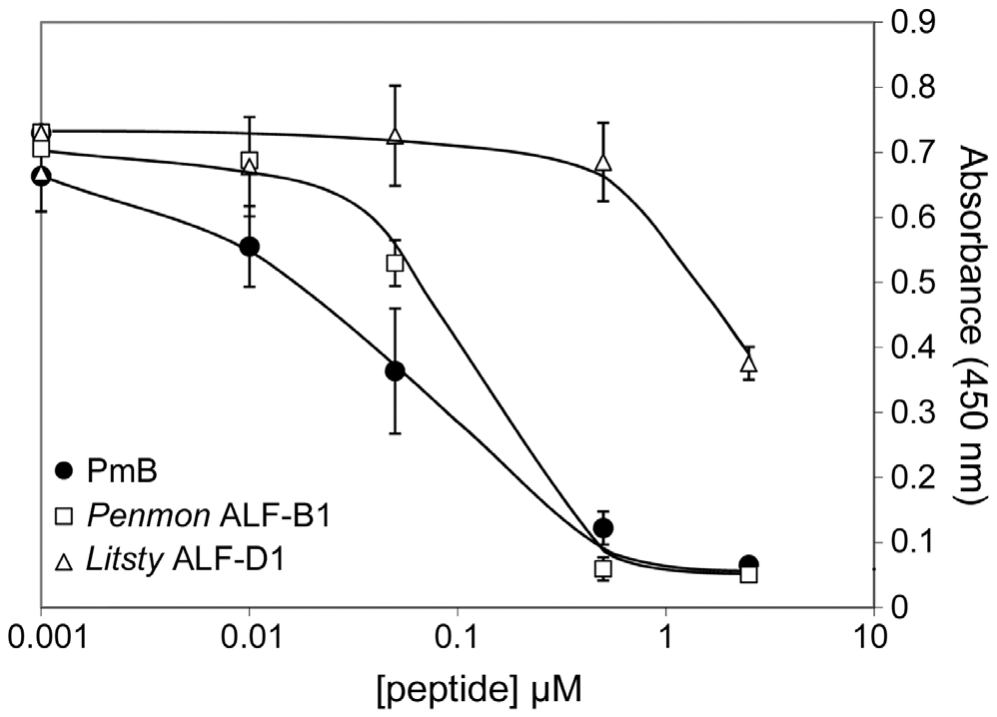
Differential LPS-binding properties of shrimp ALFs from Groups B and D. The ability of *Litsty* ALF-D1 (Group D, *open triangles*) to bind LPS was compared to that of *Penmon* ALF-B1 (Group B, *open squares*) in the *Limulus* amoebocyte lysate (LAL) assay. Polymixin B (PmB, *black circles*) was used as a positive control. Absorbance (405 nm) is indicative of LAL assay activation by LPS. LPS neutralization by LPS-binding peptides prevents LAL assay activation. It takes here 20-fold more *Litsty* ALF-D1 than *Penmon* ALF-B1 to neutralize an equivalent amount of LPS.

To investigate the functional consequences of this incomplete LPS-binding site, the Group D ALF from *L. stylirostris*, termed here *Litsty* ALF-D1, was over-expressed in *E. coli* and purified by reversed-phase HPLC. The molecular mass of the recombinant *Litsty* ALF-D1 determined by MALDI-TOF-MS (10816.9 Da) (**Figure 3**) was compatible with its calculated molecular mass (10812.3 Da). We compared *Litsty* ALF-D1, as a representative form of Group D ALFs, to the previously characterized *Penmon* ALF-B1 (also known as ALF*Pm*3) [4], [11], which belongs to the extensively studied cationic Group B ALFs. Both peptides presented highly contrasted LPS-binding properties in the *Limulus* amoebocyte lysate assay (**Figure 4**). Indeed, *Penmon* ALF-B1 bound LPS almost as efficiently as polymixin B (used as a control) with a 50% inhibition of the limulus amoebocyte lysate assay at 0.1 µM instead of 0.05 µM for PmB. In contrast, *Litsty* ALF-D1, which displayed an incomplete LPS-binding site, was 20 times less active, 2 µM being required to achieve an equivalent inhibition (**Figure 4**). Therefore, unlike the Group B *Penmon* ALF-B1, the Group D *Litsty* ALF-D1, which has an incomplete LPS-binding site, is also deficient in binding LPS.

### The Group D *Litsty* ALF-D1 has Strongly Weakened Antimicrobial Properties

Antimicrobial activities of *Litsty* ALF-D1 and *Penmon* ALF-B1 were determined by the liquid growth inhibition assay against a series of Gram-positive and Gram-negative bacterial strains as well as against filamentous fungal strains. Interestingly, *Litsty* ALF-D1 was almost inactive below 10 µM. Only one strain of *Bacillus megaterium* and one strain of *E. coli* were inhibited at 2.5 µM (**Table 3**). No activity was recorded against fungal strains. On the contrary, *Penmon* ALF-B1 was active against both bacteria and fungi with MICs as low as 0.15 µM (**Table 3**). Comparison of MICs against *E. coli* SBS363 showed that *Litsty* ALF-D1 is at least 16-fold less active that *Penmon* ALF-B1. None of the ALF tested were active against *Vibrio* strains. However, *Penmon* ALF-B1 was active against the marine strain *Aerococcus viridans* CIP 104074 at 1.25 µM. Because anionic antibacterial peptides such as *Litsty* ALF-D1 may require divalent cations for antimicrobial activity, we performed a few antimicrobial assays in the presence of sea salts (1.5 µM Ca^2+^, 5 µM Mg^2+^, 20 µM K^+^). However, *Litsty* ALF-D1 remained inactive below 10 µM against both *V. alginolyticus* and *V. harveyi* under such conditions (data not shown).

**Table 3:** Spectrum of antimicrobial activities of recombinant ALFs and synthetic ALF β-hairpins.

MIC (µM)	Recombinant ALFs		Synthetic peptides		
	*Litsty* ALF-D1	*Penmon* ALF-B1	*Litsty* ALF-D1β-hairpin	*Penmon* ALF-B1β-hairpin	*Litsty* ALF-B1β-hairpin
**Gram-positive bacteria**					
*Aerococcus viridans* CIP 104 074	>10	**1.25**	>10	**1.25**	**2.50**
*Bacillus megaterium* (IBMC collection)	**2.50**	**0.15**	>10	**1.25**	**2.5**
*Bacillus megaterium* CIP 6620	>10	**1.25**	>10	**0.60**	**1.25**
*Brevibacterium stationis* CIP 101 282	>10	>10	>10	**10**	>10
*Micrococcus luteus* CIP 5345	>10	**10**	>10	**1.25**	**1.25**
*Staphylococcus aureus* ATCC 65 38	>10	>10	>10	>10	**10**
**Gram-negative bacteria**					
*Escherichia coli* SBS363	**2.50**	**0.15**	>10	**5**	**2.50**
*Salmonella enterica* CIP 5858	>10	**2.50**	>10	**10**	**5**
*Vibrio alginolyticus* CIP 103336 T	>10	**0.60**	>10	>10	>10
*Vibrio harveyi* E22	>10	**10**	>10	>10	>10
*Vibrio anguillarum* V62	>10	>10	>10	>10	>10
*Vibrio penaeicidae* AM101	>10	>10	>10	>10	>10
*Vibrio nigripulchritudo* SFn1	>10	>10	>10	>10	>10
**Fungi**					
*Candida albicans*	>10	**10**	>10	>10	**10**
*Fusarium oxysporum*	>10	**10**	>10	**10**	**2.50**
*Rhizopus stolonifer*	>10	>10	>10	>10	**10**
*Septoria nodorum*	NT	NT	>10	**10**	**2.50**
*Botrytis cinerea*	>10	**10**	>10	>10	**10**

MIC (µM) values refer to the minimal inhibitory concentration required to achieve 100% growth inhibition. NT: not tested.

### The Antimicrobial Activity Carried by Central β-hairpin of Group B ALFs is Lacking in *Litsty* ALF-D1 (Group D)

The central β-hairpin of ALFs, which carries LPS-binding properties in many species [22]–[24], is one region poorly conserved between Group B and D compared to other groups. Indeed, 17 over the 22 residues (77.2%) of the β-hairpin sequence are identical or have conserved functions between groups B and C, 14 (63.6%) between groups B and A, and only 9 (40.9%) between groups B and D. To determine whether the central β-hairpin, could be responsible for the weak activity of *Litsty* ALF-D1, we compared the antimicrobial activity of synthetic peptides corresponding to this region in both ALF sequences. Synthetic peptides were named *Litsty* ALF-D1 β-hairpin and *Penmon* ALF-B1 β-hairpin for the anionic *Litsty* ALF-D1 and the cationic *Penmon* ALF-B1, respectively. An additional peptide was used, *Litsty* ALF-B1 β-hairpin, which corresponds to a cationic Group B ALF from *L. stylirostris.* In the liquid growth inhibition assay, no activity was recorded for *Litsty* ALF-D1 β-hairpin up to 10 µM (**Table 3**). On the contrary, both *Penmon* ALF-B1 β-hairpin and *Litsty* ALF-B1 β-hairpin, which derive from ALF Group B sequences, were active against both bacteria and fungi. They had very similar potencies and spectra of activity, the more potent activities being recorded against *B. megaterium* CIP 6620 at 0.6 and 1.25 µM, respectively (**Table 3**). Altogether, these data show that unlike the central β-hairpin of Group B ALFs, the central β-hairpin of Group D *Litsty* ALF-D1 is devoid of antimicrobial activity.

### All Four ALF Groups are Simultaneously Expressed in a Single shrimp

**Figure 5:**
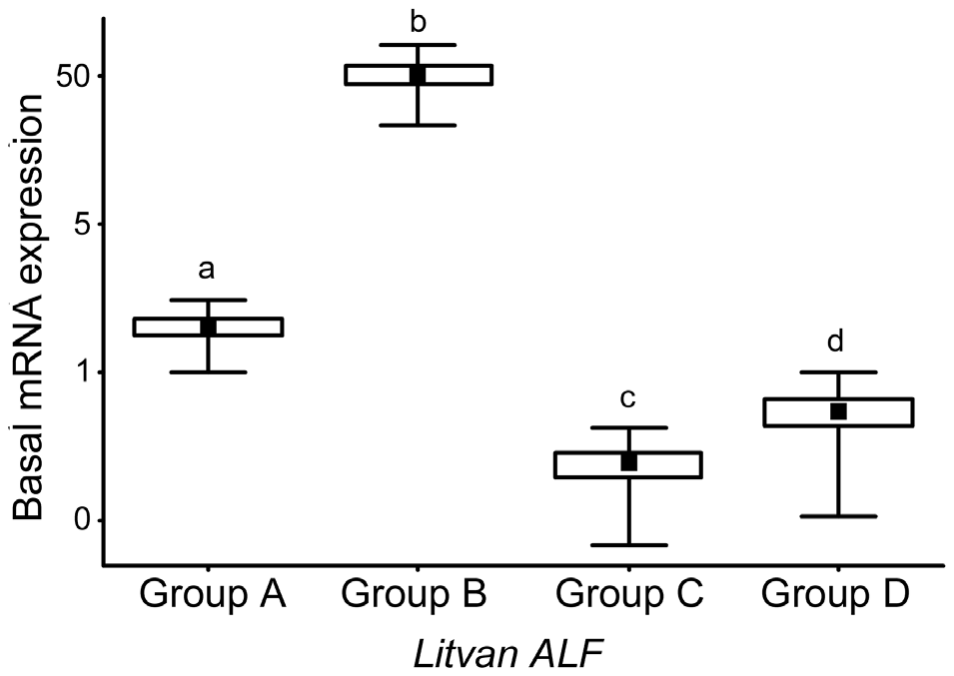
All four ALF groups are simultaneously transcribed in a single shrimp. The basal gene expression levels of the four ALF Groups identified in *L. vannamei* shrimp (*Litvan ALF-A* to *-D*) were determined by RT-qPCR on circulating hemocytes of individual shrimps (n = 15) according to the 2^−ΔΔCq^ method [32]. The ribosomal protein L40 (*Litvan-rpl40*, GenBank: FE077602) was used as a reference gene. Results are expressed as mean values (central black squares) ±SE (boxes) and ±SD (whiskers) of relative expressions on a logarithmic scale. Significant differences between the basal RNA expression levels of the four ALF Groups (Student’s t-test, *p*<0.05) are indicated by different lowercase letters (a, b, c and d). The use of a same letter indicates the absence of significant difference, while the use of different letters indicates significant difference.

Because sequences of the four ALF groups were identified in *L. vannamei* (**Figure 1**), we asked whether they could be expressed simultaneously in a single shrimp. Their expression was monitored by quantitative Real-Time PCR (RT-qPCR) in circulating hemocytes of 15 non-stimulated (naïve) *L. vannamei* shrimps. Specific primers sets were used for each ALF group (**Table 1**). Results showed that all four ALF groups are constitutively transcribed in the circulating hemocytes of a single *L. vannamei* shrimp, but at significantly different levels. *Litvan ALF-B* was the most transcribed gene, followed by *Litvan ALF-A*, *-D* and *-C* (**Figure 5**). The basal gene expression levels of *Litvan ALF-B* was 49-fold (*p*<0.0001), 253-fold (*p*<0.0001) and 361-fold (*p*<0.0001) higher than *Litvan ALF-A*, *-D* and *-C*, respectively. Comparatively, the basal mRNA expression of *Litvan ALF-A* was 5.2-fold (*p*<0.0001) and 7.4-fold (*p*<0.0001) higher than *Litvan ALF-D* and *-C*, respectively (**Figure 5**). Although we found a significant difference between the basal mRNA expression of *Litvan ALF-C* and *Litvan ALF-D* (*p*<0.002), it was not possible to determine which of these two genes is the most expressed due to the small differences in gene expression (<2-fold change) and the use of primer pairs with different efficiencies (E = 2±0.2).

### ALF Groups are Differentially Regulated in Response to a Microbial Challenge

**Figure 6:**
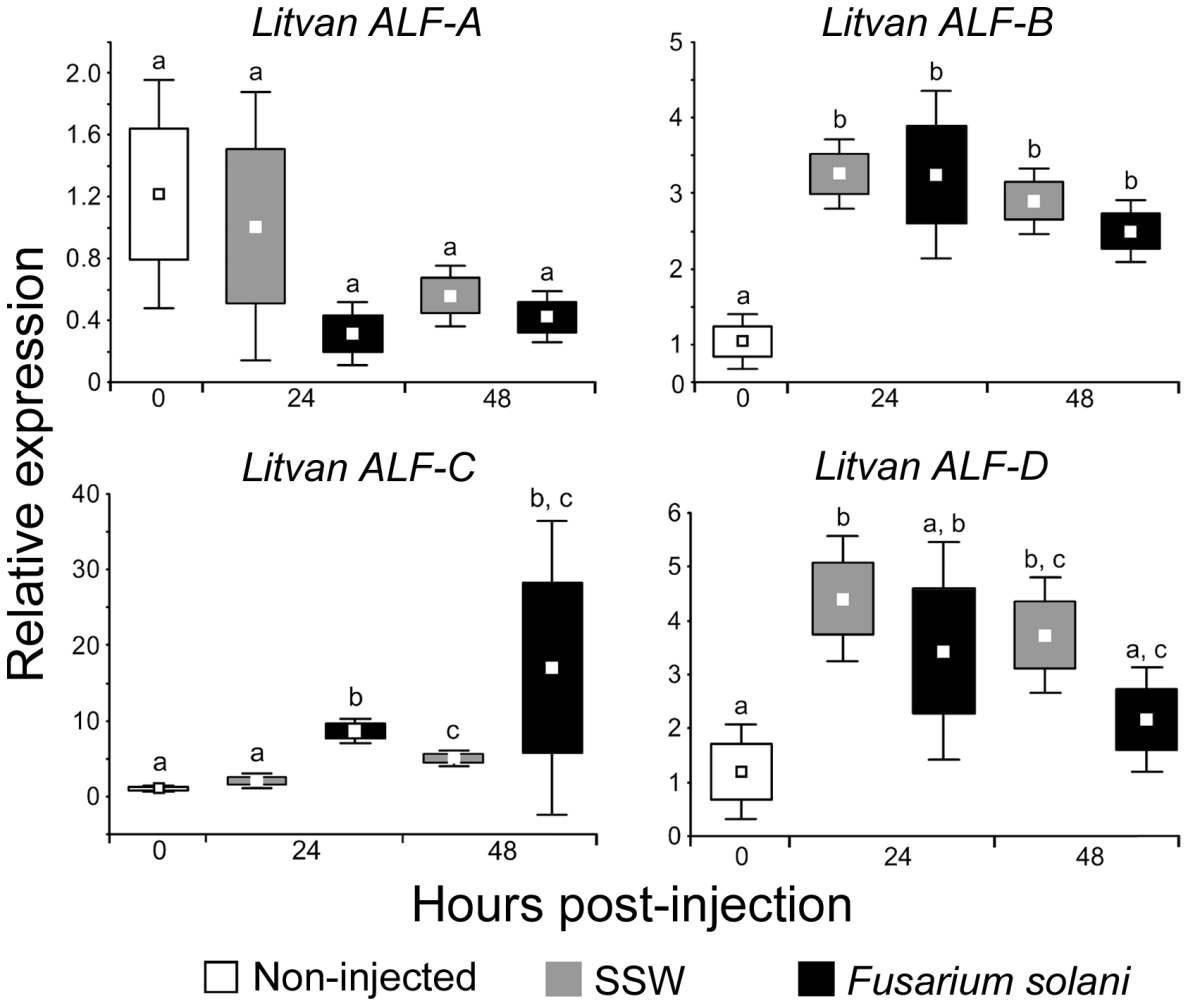
Differential expression of ALF groups in response to an experimental infection. The transcript abundance of the four ALF Groups (*Litvan ALF-A*, *-B*, *-C*, and *-D*) was measured by RT-qPCR on circulating hemocytes of shrimp (three groups of three shrimps per condition) injected with the shrimp pathogen *Fusarium solani* (black boxes), sterile sea water (SSW, grey boxes) or non-injected (white boxes). Analyses were performed 24 and 48 hours after injection. Relative abundances of ALF transcripts are calculated according the 2^−ΔΔCq^ method [32], with the *L. vannamei* ribosomal protein L40 (*Litvan-rpl40*, GenBank: FE077602) used as reference gene. Results are expressed as mean values (central squares) ±SE (boxes) and ±SD (whiskers) of relative expressions. Significant differences between conditions for each ALF groups (Student’s t-test, *p*<0.05) are indicated by different lowercase letters (a, b, and c). The use of a same letter indicates the absence of significant difference, while the use of different letters indicates significant difference.

The functional diversity of shrimp ALFs evidenced here, led us ask how these genes respond to an injury or to a microbial infection. For that, we analyzed by RT-qPCR the expression profile of ALF groups in circulating hemocytes of shrimp injected with sterile sea water (SSW) or infected with the fungal pathogen *Fusarium solani* [31]. Infections were performed in the shrimp *L. vannamei* for which ALF sequences from all four groups were available in the databases. Every group of ALFs responded differently to the microbial challenge (**Figure 6**). The transcript abundance of *Litvan ALF-A* did not differ between shrimp injected with *Fusarium* or SSW and non-injected shrimp. On the contrary, the transcript abundance of *Litvan ALF-B*, *-C*, and *-D* followed different expression profiles. Thus, after an injection of SSW or *Fusarium*, the abundance of *Litvan ALF-B* transcripts was 2.4 to 3.1 fold higher than in non-injected shrimp (48 h after *Fusarium* injection and 24 h after SSW injection, respectively). No significant difference was observed between 24 h and 48 h. Conversely, the transcript abundance of *Litvan ALF-C* varied with the type of injection. Thus, after 24 h, only shrimp injected with *Fusarium* showed a *Litvan ALF-C* transcript abundance 8.1 fold higher than non-injected shrimp. However, after 48 h, it was 4.8 fold and a 16 fold for SSW and *Fusarium*-injected shrimp, respectively. Finally, for *Litvan ALF-D*, only shrimp injected with SSW had significantly higher transcript abundance, from 3.7 fold (24 h) to 3.1 fold (48 h), than non-injected shrimp.

## Discussion

Results showed that shrimp ALFs cluster into four groups diverse in terms of primary sequence, biochemical properties, biological activities and gene expression. We provide here the first evidence of functional divergence in shrimp ALFs. The novel members of the ALF family identified in this study form a phylogenetic group termed Group D distinct from the previously characterized Groups A, B and C. Group D ALFs have a negative net charge (p*I* ≤6.2) which contrasts with the most common properties of eukaryotic antimicrobial peptides, whose positive net charge mediates interaction with the electronegative membranes of bacteria [36]. In addition, Group D ALFs display only two conserved residues out of seven charged residues [Glu^21^, Lys^22^, (Lys/Arg)^31^, Lys^35^, (Lys/Arg)^46^, (Lys/Arg)^48^, (Lys/Arg)^58^] from the ALF β-sheet proposed to be involved in the LPS-binding site of *Penmon* ALF-B1 [11]. The other five residues are substituted by uncharged amino acids (**Figure 1**). We showed here that such substitutions impaired the interaction of Group D ALFs with LPS. Indeed, Group D *Litsty* ALF-D1 produced here as a recombinant peptide was 20-fold less effective in binding LPS than our reference recombinant Group B ALF, *Penmon* ALF-B1 (**Figure 4**). Together with previous biochemical data on the horseshoe crab ALF [24] and our previous molecular modeling data based on the shrimp *Penmon* ALF-B1 NMR structure [11], this strongly supports the role of the charged residues carried by the four-stranded β-sheet of ALFs in their interaction with the hydrophilic and phosphate groups of lipid A. Importantly, *Litsty* ALF-D1, which displays a poor conservation of the putative LPS-binding residues, was also poorly active against Gram-negative bacteria with MIC values at least 16-fold higher than the Group B *Penmon* ALF-B1 (**Table 3**). The same was true for the synthetic β-harpin region of *Litsty* ALF-D1, which lacked antibacterial activity, as opposed to the synthetic β-harpin regions of *Litsty* ALF-B1 and *Penmon* ALF-B1 **(** **Table 3** **).** Therefore, LPS-binding appears essential for the anti-Gram-negative activity of shrimp ALFs.

Altogether, our phylogenetic and functional data indicate that ALFs have evolved as functionally diverse groups, among which some have selected important motifs for interacting with LPS. Our present study demonstrates the very intimate link existing between such recognition functions and the subsequent antimicrobial properties of ALFs. Together with our previous study on oyster defensin functional diversity [29], this shows how far sequence diversity affecting amino acids involved in the recognition of microbe cell wall components has shaped the activity of marine invertebrate antimicrobial peptides and led to functional divergence.

Because *Litsty* ALF-D1 was only poorly active against Gram-negatives, we asked whether it had evolved towards other antibacterial functions. However, *Litsty* ALF-D1 was not active against filamentous fungi and only poorly active against Gram-positive bacteria as compared to our broad-spectrum Group B reference (**Table 3**). Similarly, synthetic peptides corresponding to the central β-hairpin of Group D ALFs were devoid of anti-Gram-positive activity, as opposed to peptides designed on the same region of Group B ALFs (**Table 3**). This is in agreement with the important role of the central β-hairpin in the anti-Gram-positive activity of Group B ALFs [4]. It also tells that the major reduction of the positive net charge in this functional region may not only impair the electrostatic interaction with the LPS of Gram-negative bacteria but also with LTA, a major cell wall component of Gram-positive bacteria previously reported to bind to Group B ALFs [19]. Because some Group A ALFs also display a negative net charge [13], but have a more conserved β-harpin sequence, investigations on their antibacterial properties should help understanding whether conserved amino acids interacting with specific cell wall components or a global positive charge are required for the antibacterial activity of shrimp ALFs.

From our *in silico* analysis (**Figure 2**), Group D ALFs would be only found in occidental shrimp, such as *L. vannamei*, *L. setiferus* and *L. stylirostris*. This is reminiscent of penaeidins, another major AMP family of penaeid shrimp. Indeed, while *PEN2*, *PEN3* and *PEN4* are found in occidental shrimp, only *PEN3* and *PEN5* are found in Asian shrimps [13]. Therefore, for those two main families of shrimp AMPs, genes encoding AMP groups appear to be differentially distributed among Asian and occidental shrimp species.

By using *L. vannamei* to conduct our expression study, we showed here that all four members of the ALF family (A, B, C and D) are simultaneously expressed in a single shrimp. This was also shown for penaeidin genes (*PEN2*, *PEN3* and *PEN4*) from the same species [37]. Interestingly, as in *P. monodon* [16], Group B ALFs were by far the most transcribed ALF genes in *L. vannamei* shrimp (by more than 50 fold; **Figure 5**). The high expression and the highly potent antimicrobial activity of Group B ALFs (**Table 3**) suggest they likely play an important protective role in the shrimp antimicrobial defense. Whereas there is no evidence of ALF expression at the protein level, RNA interference data strongly support the major role of *Penmon* ALF-B1 in shrimp antibacterial and antiviral defense [14], [26].

Group A and C ALFs, which were shown here to be expressed at much lower levels than Group B ALFs (**Figure 5**), would also display important functions in shrimp survival to both bacterial and fungal infections [14], [25]. Since all groups of antimicrobial ALFs are expressed simultaneously in a single shrimp (**Figure 5**), it can be hypothesized that the high sequence diversity of shrimp ALFs contributes to create synergism improving shrimp antimicrobial defenses [12]. Such a synergism has been previously demonstrated for variants of oyster defensins [29], [38].

The role of Group D ALFs in shrimp defense remains much more ambiguous. On the one hand, *Litsty* ALF-D1 was shown to be poorly antimicrobial (**Table 3**). On the other hand, expression of *Litsty ALF-D1* is not correlated with the capacity of *L. stylirostris* shrimp to survive a pathogenic *Vibrio* infection [7]. Therefore, unlike other ALF groups, Group D ALFs would not play a direct antibacterial/antifungal functions in shrimp defense but could have evolved towards uncharacterized functions (neofunctionalization). Indeed, it is now well established that AMPs are multifunctional peptides whose defense functions go far beyond their antimicrobial properties [39]. One interesting function for shrimp defense that remains to be investigated is the antiviral activity of this ALF group.

Finally, while the gene expression profile of ALFs has been mainly investigated in response to viruses and bacteria challenges [13], we analyzed here the transcript abundance of the four *Litvan ALF* genes in shrimp infected with the fungal pathogen *F. solani*. Interestingly, shrimp ALFs were shown to follow different gene regulation patterns in response to a fungal infection. Thus, while *Litvan ALF-A* is not modulated, *Litvan ALF-B*, -*C*, and *-D* are induced in circulating hemocytes in response to *F. solani* challenge and to injury (**Figure 6**). Such a differential regulation has been earlier observed among variants of other AMP families such as the oyster big defensins (*Cg*-BigDefs) and human β-defensins (HBD) [40], [41]. Together with important sequence variations between ALF groups, this suggests that different genes encode ALFs from different groups in *L. vannamei*. This is in agreement with data on *P. monodon* showing that each of the three ALFs (Group A to C) is encoded by a distinct genomic sequence [14], [16]. The existence of different genetic locations for ALF groups supports further the functional divergence of this family of AMPs, as also proposed for oyster defensins [42].

To conclude, taking into account the high diversity of shrimp ALFs explored here, we propose a uniform nomenclature for shrimp ALFs, as adopted for other AMP families, such as avian β-defensins [43], amphibian skin peptides [44], and shrimp penaeidins [35]. Like the penaeidin (PEN) nomenclature, the shrimp ALF nomenclature is based on the conserved amino acid residues found in the mature polypeptides of each ALF Group (**Figure 1**). For instance, in *L. vannamei* shrimp, the described ALF variants were named as ALF*Lv*1 to -3 by Jiménez-Vega and Vargas-Albores [45] or *Lv*ALF1 and -2 by de la Vega *et al*. [25]. The aim of this recommended nomenclature is to standardize the confusing terminology currently employed for this highly diverse family of shrimp antimicrobial peptides [13], [17], [18], [22], [46].

## Supporting Information

Table S1 **Sequences and biochemical properties of shrimp ALFs.** The names of ALF sequences (groups A to D) are given in the new nomenclature. The corresponding ancient names are also provided together with existing GenBank numbers. Amino acid sequences of signal peptides and mature peptides are displayed with the number of amino acids and theorical pI of the polypeptides.
(XLSX)
